# Immunomodulatory Function of Myeloid-Derived Suppressor Cells during B Cell-Mediated Immune Responses

**DOI:** 10.3390/ijms19051468

**Published:** 2018-05-15

**Authors:** Bilgenaz Özkan, Heejin Lim, Sung-Gyoo Park

**Affiliations:** School of Life Sciences, Gwangju Institute of Science and Technology, Gwangju 61005, Korea; bilgenazozkan@gist.ac.kr (B.Ö.); heejin425@gist.ac.kr (H.L.)

**Keywords:** MDSCs, B cells, inflammation, M-MDSCs, PMN-MDSCs

## Abstract

Myeloid-derived suppressor cells (MDSCs) play roles in immune regulation during neoplastic and non-neoplastic inflammatory responses. This immune regulatory function is directed mainly toward T cells. However, MDSCs also regulate other cell populations, including B cells, during inflammatory responses. Indeed, B cells are essential for antibody-mediated immune responses. MDSCs regulate B cell immune responses directly via expression of effector molecules and indirectly by controlling other immune regulatory cells. B cell-mediated immune responses are a major component of the overall immune response; thus, MDSCs play a prominent role in their regulation. Here, we review the current knowledge about MDSC-mediated regulation of B cell responses.

## 1. Introduction

Myeloid-derived suppressor cells (MDSCs) were first described in a lung cancer mouse model system in 1987 [1]. Since then, many papers have described the function of MDSCs in both mouse models of cancer [2,3] and in cancer patients [4,5]. In addition, MDSCs are also involved in virus-mediated inflammatory responses, non-neoplastic inflammatory responses, and autoimmune responses [6,7,8]. Nowadays, it is thought that MDSCs comprise a heterogeneous population of immature myeloid cells [9]. During inflammatory responses, MDSCs accumulate and become activated to directly or indirectly regulate innate and adaptive immune responses [10]. MDSCs suppress immune responses by producing reactive oxygen species (ROS), peroxynitrite (PNT), and anti-inflammatory cytokines [7,11]. In addition, these populations also suppress T cell responses directly via interaction between programmed cell death-1 (PD-1) and its ligand (PD-L1) [12]. MDSCs also regulate immune responses indirectly by controlling differentiation of regulatory T (Treg) cells and regulatory dendritic cells [13,14]. Recent findings reveal bidirectional regulation between Treg cells and MDSCs [13]. Natural Killer cells (NK cell) are also regulated by MDSCs in different ways. Tumor growth factor-β (TGF-β) secreted by M-MDSCs suppresses NK cell function by decreasing IFNγ production by NK cells [9,15]. Additionally, ROS and prostaglandin E2 (PGE2) secreted by M-MDSCs from cancer patients also suppress NK cell function [15]. The immunosuppressive functions of MDSCs are directed mainly at T cells; however, reports suggest that they also regulate B cell immune responses, DC-mediated immune responses, and macrophage-mediated immune responses [16,17,18].

Here, we discuss the role of MDSCs during different stages of B cell immune responses (e.g., B cell differentiation and B cell activation and antibody production). We also discuss the molecular mechanisms underlying MDSC-mediated regulation of B cells.

## 2. General Phenotype of MDSCs

MDSCs are a heterogeneous population of immature myeloid cells. However, MDSCs can be divided into two populations: monocytic MDSCs (M-MDSCs) and polymorphonuclear MDSCs (PMN-MDSCs). Morphologically speaking, M-MDSCs are similar to monocytes, whereas PMN-MDSCs have multi-lobed nuclei similar to those of PMN cells [7,19]. Here, we follow the nomenclature used in the original paper, i.e., PMN-MDSCs. The two populations also express different surface molecules; thus, they can also be subdivided on this basis [20]. In mice, M-MDSCs and PMN-MDSCs are defined as CD11b^+^Gr-1^+^Ly6G^low^Ly6C^high^ and CD11b^+^Gr-1^+^Ly6G^high^Ly6C^low^, respectively. However, human MDSCs lack Gr-1 expression [19]. Currently, human MDSCs are defined according to expression of CD33, CD11b, HLA-DR, CD14, and CD15 [21,22]; therefore, the phenotype of human M-MDSCs is CD33^+^CD11b^+^HLA-DR^low/−^CD14^+^CD15^low/−^ and that of human PMN-MDSCs is CD33^+^CD11b^+^HLA-DR^low/−^CD14^−^CD15^+^CD66b^+^. Although the role of each population in cancer and inflammatory responses remains unclear, predominance of certain subsets of MDSCs differs between different cancers. M-MDSCs are dominant in brain, ovarian, and prostate cancers, and in lung and hepatocellular carcinoma [23,24,25,26], whereas PMN-MDSCs are dominant in head and neck cancer [27]. 

## 3. MDSC-Mediated Regulation of B Cell Differentiation

A previous study shows that adipocyte-derived factors such as fatty acids, free cholesterol, ceramides, and lipid crystals regulate B cell differentiation [28]. In addition, adipocyte-derived factors promote generation of MDSCs by acting as danger-associated molecular patterns, which trigger activation of the inflammasome in MDSCs [29,30]; this leads ultimately to increased production of IL-1β by MDSCs [29,31]. Kennedy and Knight revealed that MDSCs have the potential to inhibit B lymphopoiesis because B lineage cells did not develop in an in vitro model of B lymphopoiesis in the presence of CD11b^hi^Gr1^+^ MDSCs isolated from adipocyte-conditioned medium-treated bone marrow cultures [29]. They also showed that IL-1 produced by MDSCs inhibits differentiation of multipotent progenitors into B lineage cells. In addition, although MDSCs usually suppress proliferation of T cells by secreting inducible nitric oxide synthase (iNOS) and arginase, these mechanisms do not play a role in B lymphopoiesis [29]. Treatment with IL-1 also increases the number of CD11b^+^Gr1^+^ myeloid cells in culture by promoting myelopoiesis at the multipotent progenitor stage [29,32].

## 4. Direct Regulation of B Cell Responses by Effector Molecules Expressed by MDSCs

**Figure 1:**
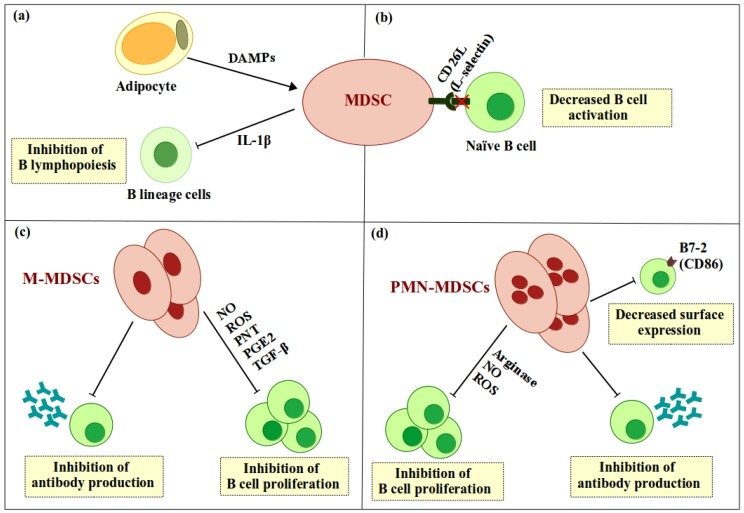
Direct regulation of B cells by myeloid-derived suppressor cells (MDSCs): (**a**) Adipocyte-induced accumulation of MDSCs inhibits development of B lineage cells via secretion of interleukin 1β (IL-1β). (**b**) MDSCs downregulate surface expression of the adhesion molecule L-selectin (CD62L) on B cells, which in turn inhibits lymphocyte homing to activation sites. (**c**) M-MDSCs inhibit B cell proliferation and antibody production (IgM and IgG) by secreting arginase, nitric oxide (NO), reactive oxygen species (ROS), peroxynitrite (PNT), prostaglandin E2 (PGE2), and tumor growth factor-β (TGF-β). (**d**) Polymorphonuclear MDSCs (PMN-MDSCs) inhibit B cell proliferation and antibody production (IgM) by secreting arginase, NO, and ROS. 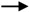, positive effect; 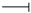, negative effect.

Although studies show that MDSCs suppress B cell function, the underlying mechanisms and effector molecules involved are unclear. Studies using specific inhibitors revealed that MDSC-derived arginase-1, nitric oxide (NO), ROS, TGF-β, and PGE2 play roles in MDSC-mediated suppression of CD19^+^ B cell proliferation [16,17,33]. In addition, Lelis and colleagues demonstrated that human PMN-MDSCs induce B cell necrosis in a contact-dependent manner [33] (Figure 1).

### 4.1. Arginase-1

l-arginine is the substrate for arginase-1 and iNOS enzymes, both of which are expressed at high levels by MDSCs; these molecules play a role in direct MDSC-mediated suppression of T cell function by depleting l-arginine from the microenvironment and by generating NO, respectively [34,35]. While M-MDSC-mediated suppression of B cell responses by NO has been reported, arginase-1-dependent B cell regulation by M-MDSCs has not been tested [36,37]. M-MDSC-mediated suppression of B cell responses in LB-BM5 retroviral-infected mice is independent of arginase activity since suppression of B cell responses is not affected by addition of an arginase-specific inhibitor, Nor-NOHA, to suppression assays [17,37]. In contrast to M-MDSC-mediated suppression, another study added an arginase-specific inhibitor, Nor-NOHA, to co-cultures of human PMN-MDSCs and B cells, thereby identifying arginase-1 as a potential factor involved in human PMN-MDSC-mediated suppression of B cell proliferation [33].

### 4.2. NO/ROS

Increased NO production by MDSCs via upregulation of iNOS activity is one of the main mechanisms by which they suppress T cell function by inducing T cell apoptosis or by inhibiting phosphorylation of signaling proteins such as Janus kinase 3 or signal transducer and activator of transcription 5 (Stat5), both of which act downstream of the IL-2 receptor [38,39]. To the best of our knowledge, Crook and colleagues demonstrated for the first time that MDSCs suppress B cell function during autoimmune disease [16]. A study in a collagen-induced arthritis (CIA) mouse model showed that co-culturing CD40L/IL-4-stimulated B cells with M-MDSCs increased NO levels in the supernatants of cell cultures. Moreover, the use of iNOS inhibitors (to reverse the increased levels of NO) abolished the suppressive effect of M-MDSCs on B cell proliferation in co-cultures of M-MDSCs and B cells. However, addition of iNOS inhibitors had no effect on NO levels in cultures of B cells alone, or in co-cultures of B cells and Ly6G^+^ cells [16]. Green and colleagues characterized the suppressive effects of M-MDSCs on B cell responsiveness in a LP-BM5 retroviral infection system [37]. They used a LP-BM5 retrovirus-infection mouse model to show that M-MDSCs suppressed T cell responses in an iNOS-dependent manner, whereas B cell responses to LPS, anti-CD40, and IL-10 were only partially dependent on the iNOS pathway. Moreover, NO production correlated with the suppressive effect of M-MDSCs on B cells. Although NO production by M-MDSCs was completely inhibited by an iNOS inhibitor, suppression assays revealed that NO inhibition had only a partial impact on B cell responses [17]. Thus, M-MDSC-mediated inhibition of B cell responses is not completely dependent on the NO/iNOS system.

Transwell assays revealed that M-MDSCs were capable, at least partially, of mediating suppression of B cell activation in the absence of cell–cell contact, hinting at a role for secreted factors in M-MDSC mediated suppression of B cell responses. A Griess assay performed as a part of Transwell experiment system revealed that approximately half of M-MDSC-mediated B cell suppression was due to iNOS-mediated production of NO, whereas approximately 80% was cell-to-cell contact independent [17]. Thus, the authors suggested one or more contact-independent mechanisms underlying M-MDSC-mediated suppression of B cell proliferation [17,37]. However, when Rastad and Green exposed B cells to supernatant taken from suppressive M-MDSCs, they found that suppression of B cell responses was not complete; therefore, they suggested that a contact-dependent mechanism also contributes to MDSC-mediated suppression of B cell activity [17].

In addition to the increased NO production, one of the main characteristics of MDSCs is increased production of ROS, which in several mouse tumor models and human cancer patients is induced by tumor-derived factors [6,40]. Increased production of ROS and PNT by MDSCs inhibits T cell responses by abolishing their ability to recognize antigens; this occurs via nitration of amino acids on the T cell surface, which occurs during interaction between MDSCs and T cells [41]. ROS such as hydrogen peroxide and superoxide are soluble mediators involved in MDSC-mediated T cell suppression; indeed, MDSCs induced accumulation of hydrogen peroxide through arginase activity in tumor growth [40]. Involvement of hydrogen peroxide in M-MDSC-mediated B cell suppression was tested by adding catalase, which converts hydrogen peroxide to water and molecular oxygen, to the M-MDSC supernatant; this was then added to B cell cultures. The results showed that B cell responses were still affected by the transferred supernatant [17,42]. Moreover, to test the involvement of superoxide as another ROS, superoxide dismutase (SOD), which induces conversion of superoxide to hydrogen peroxide, was added to the supernatant transfer assays; the results revealed only partial suppression of B cell responses [17]. Subsequently, results obtained by XTT (2,3-bis-(2-methoxy-4-nitro-5-sulfophenyl)-2H-tetrazolium-5-carboxanilide) assays performed in parallel with the supernatant transfer assays supported the former results, since superoxide was only detected in the supernatant of B cell/M-MDSC co-cultures but not in control supernatants from B cells alone [17].

The reaction between superoxide anion and NO in myeloid cells generates a more potent oxidant called PNT [43], which is one of the soluble mediators involved in M-MDSC-mediated suppression of B cell responsiveness via the iNOS pathway [37]. Rastad and Green used uric acid and MnTBAP as PNT scavengers to confirm that MDSC-mediated blockade of B cell function depends, at least in part, on PNT in a dose-dependent manner. In addition to the role of NO in forming PNT, they used carboxy-PTIO (a specific scavenger of NO) to demonstrate that it was involved in M-MDSC-mediated suppression of B cell responses in a dose-dependent manner. Furthermore, the suppressive effect of M-MDSCs was tested by checking the additive effect of superoxide and NO by blocking each with SOD and L-Nil, respectively. The suppressive effect of M-MDSC supernatants in the presence of SOD and L-Nil was significantly lower than that of M-MDSC supernatants containing each inhibitor alone, suggesting that superoxide and NO are generated by non-overlapping pathways [17].

### 4.3. TGF-β

TGF-β is a cytokine involved in several cellular events, including proliferation, survival, and migration; increased secretion of TGF-β by MDSCs promotes tumor progression by suppressing T cell proliferation [44]. Rastad and Green examined soluble TGF-β as a potential inhibitor of B cell responses since MDSC supernatant significantly inhibited B cell proliferation, a phenomenon mediated mainly by NO, superoxide and PNT [17]. However, suppression was partly dependent on soluble TGF-β released by M-MDSCs because an anti-TGF-β antibody had a significant effect on MDSC-mediated suppression of B cell responses [17].

### 4.4. PGE2

PGE2 plays a role in tumor progression by increasing expression of PGE2 receptors on MDSCs and by inducing bone marrow stem cells to differentiate into Gr1^+^CD11b^+^ MDSCs [45]. PGE2 inhibits differentiation of monocytes into dendritic cells by inducing expression of cyclooxygenase 2; it also induces expression of suppressive factors by MDSCs, such as IL-4Rα, NOS2, IL-10, and arginase [46,47]. PGE2 interferes with the early stages of B cell activation [48]. Crook and colleagues examined involvement of PGE2 in MDSC-mediated suppression of B cells in CIA model mice by co-culturing B cells with M-MDSCs and Ly6G^+^ cells. They found that PGE2 levels increased in the supernatant when B cells were co-cultured with M-MDSCs but not when B cells or M-MDSCs were cultured alone, or when B cells were co-cultured with Ly6C^+^ cells. Co-culture of B cells and M-MDSCs revealed that M-MDSC-mediated suppression of B cell proliferation was reduced when PGE2 was prevented from binding to PGE2 receptors by EP2 and EP4 (which are PGE2 receptor antagonists) [16]. Thus, M-MDSC-mediated suppression of B cell proliferation requires PGE2 [16].

### 4.5. Cysteine

Increasing cysteine concentrations (by using β-mercaptoethanol to reduce extracellular cystine to cysteine) has no effect on suppression of B cell responses by M-MDSCs, even though cysteine depletion is one mechanism by which MDSCs mediate T cell suppression [17,49]. 

## 5. Direct Regulation of B Cell Responses by MDSCs via Expression of Cell Surface Molecules

Crook and colleagues [16] demonstrated that proliferation of CD40L/IL-4-stimulated B cells in a CIA mouse model was suppressed significantly by autologous M-MDSCs in a concentration-dependent manner when CD40L/IL-4-stimulated B cells isolated from the spleen were co-cultured with M-MDSCs and Ly6G^+^ cells isolated from the bone marrow. In addition, they showed that MDSCs suppress B cell function by decreasing antibody production in vitro and by decreasing serum levels of antigen-specific antibodies in a CIA mouse model [16]. Lelis and colleagues also demonstrated that MDSCs modulate antibody production by B cells, since IgM production by B cells induced with anti-IgM F(ab`)_2_/CpG was significantly blocked by PMN-MDSCs in a dose-dependent manner [33].

Data from their suppression assays conducted in Transwell system suggest that the suppressive effect of MDSCs on B cells requires cell-to-cell contact since suppression of B cells by M-MDSCs was reduced when they were separated by the Transwell [16]. Moreover, they revealed that M-MDSC-mediated suppression of B cell responses via NO and PGE2 also requires cell-to-cell contact since increased NO and PGE2 production by M-MDSCs was abolished when M-MDSCs and B cells were separated by the Transwell [16]. Lelis and colleagues demonstrated the suppressive effect of human PMN-MDSCs on activation of specific B cells by anti-IgM F(ab`)_2_ and ODN CpG, whereas they had no effect on proliferation of B cells activated with PMA/ionomycin. They also used Transwell assays to demonstrate that cell-to-cell contact is required for human PMN-MDSC-mediated suppression of B cells via arginase-1, NO, and ROS, but independent of indoleamine-2,3-dioxygenase. In addition, they revealed that human PMN-MDSCs induce B cell death in a contact-dependent manner [33]. Thus, unknown surface molecules on these cells mediate MDSC-mediated suppression of B cell function.

Human PMN-MDSCs also reduce surface expression of the costimulatory molecule B7-2 (CD86) on the surface of B cells activated by anti-IgM F(ab`)_2_/CpG in a dose-dependent manner. The B7-2 molecule is important since it is upregulated on activated B cells and upon engagement it induces formation of germinal centers by activating follicular helper T cells, which are themselves important for activation of T cells and for proliferation and differentiation of B cells [33,50]. Thus, Lelis and colleagues suggested that human PMN-MDSCs modulate B cell activation pathways by reducing expression of B7-2 [33].

l-selectin (or CD62L), a carbohydrate-binding cell adhesion molecule expressed by lymphocytes, regulates leukocyte trafficking by directing naïve lymphocytes to sites of activation [51,52]. MDSCs reduce the ability of naïve T cells to home to activation sites by decreasing l-selectin expression by T cells through ADAM17-mediated cleavage of the ectodomain region of l-selectin [53]. However, tumor-induced MDSCs downregulate expression of l-selectin on murine CD4^+^ and CD8^+^ naïve T cells and on blood-borne murine B cells in a contact-dependent manner that is independent of ADAM17 [54]. Thus, an as yet unknown molecule on the surface of MDSCs plays a role in downregulating l-selectin on B cells. In addition, MDSC-mediated loss of l-selectin from B cells was not detected in bone marrow and splenic compartments, whereas l-selectin expression on blood-borne B cells was almost completely downregulated; thus, loss of l-selectin from B cells is differentially regulated by sub-anatomical compartments in tumor-bearing mice [55].

In addition to iNOS-mediated mechanisms, Green and colleagues demonstrated that V-domain Ig suppressor of activation (VISTA) also has a major effect on M-MDSC-mediated suppression of B cell responsiveness [54]. VISTA is a novel negative checkpoint ligand homologous to PD-L1, which suppresses activation of T cells [56]. An anti-VISTA monoclonal antibody blocked MDSC-mediated suppression of B cells responses, but not that of T cell responses. MDSC-mediated suppression on B cell responses was blocked completely in their experimental system when both iNOS/NO and VISTA were inhibited simultaneously [54]. Studies in iNOS/VISTA double-knockout mice reveal that suppression of B cell responses by M-MDSCs is mediated in a manner not significantly different from that in wild-type mice. In addition, M-MDSC-mediated suppression of B cell responses in iNOS/VISTA double-knockout mice is not affected by addition L-Nil (an iNOS inhibitor). Based on these data, Rastad and Green suggested that other minor mechanisms can compensate for the suppressive effect of M-MDSCs in vivo, even in the absence of the two dominant suppressive mechanisms (iNOS and VISTA) [17].

## 6. Indirect Regulation of B Cell Responses by MDSCs

### 6.1. Regulatory B Cells

Regulatory B (Breg) cells are a type of B cell that releases IL-10 and has an immunosuppressive function [57,58]. Since Breg cells play a role in some diseases and regulate other immune cells, understanding Breg cells is of increasing importance [59]. Therefore, several studies have been conducted to examine the origin of Breg cells, the different Breg subsets, and the cells that they target [57,58,59,60]. Breg cells target T cells, antigen presenting cells, NK cells, and B cells by inducing immune tolerance via production of immune regulatory cytokines such as IL-10, TGF-β, and IL-35 [59]. However, unlike Treg cells, there is no known Breg-cell-specific transcription factor; this is (partially) due to the heterogeneity of Breg cells [57]. In addition, some speculate whether Breg cells are derived from a single progenitor, called the transitional 2 marginal-zone precursor cell [58], or whether any B cell has the potential to differentiate into a Breg cell [57].

**Figure 2:**
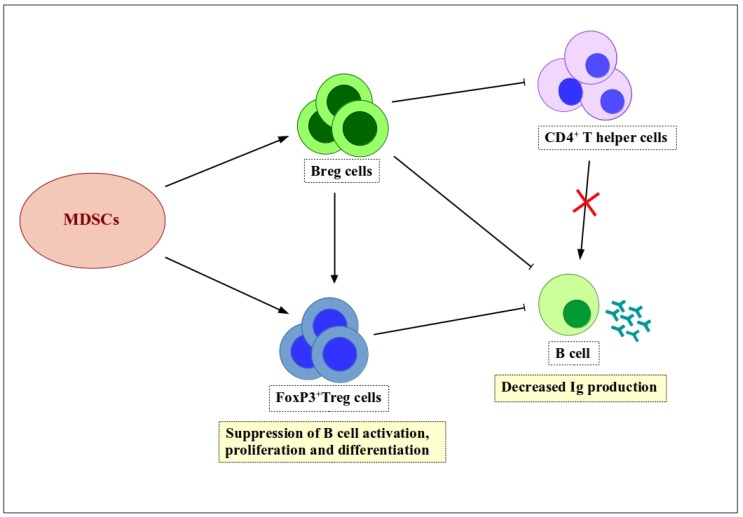
Indirect regulation of B cell functions by MDSCs via expansion of Breg and Treg cells. MDSCs induce expansion of Breg and Treg cells, both of which have suppressive characteristics. Breg cells suppress Ig production by B cells either directly or indirectly by increasing the number of Treg cells. In addition, Breg cells suppress B cell activation by downregulating CD4^+^ T helper cells. Treg cells suppress antibody production by B cells, as well as inhibiting B cell activation, proliferation, and differentiation. 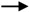, positive effect; 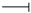, negative effect.

As mentioned before, Breg cells regulate B cell functions. More specifically, they regulate antibody production by effector B cells [59,61,62]. Although the underlying mechanism is unknown, research suggests three possible alternatives: direct suppression by Breg cells, or indirect suppression by reducing the helper CD4^+^ T cell population or increasing the Treg cell population [59]. M-MDSCs from LP-BM5-infected mice reduce the amount of IL-10 produced by Breg cells in response to LPS-stimulation [17]. Park et al. used a systemic lupus erythematosus (SLE) mouse model to assess the positive effects of MDSCs on Breg cells [63]. They concluded that MDSCs induce expansion of Breg cells via iNOS and ameliorate autoimmunity [63]. Consequently, it is possible to say that MDSCs suppress B cell responses indirectly via a mechanism involving expansion of Breg cells (Figure 2).

### 6.2. Treg Cells

Treg cells also exert an immunomodulatory function, and suppress B cell-mediated immunoglobulin production [64,65,66] as well as B cell activation [64,65,66,67], proliferation [64,66,67] and differentiation [66]. Lim et al. suggested that Treg cells suppress B cell Ig production directly and inhibit class switch recombination [64]. Iikuni and colleagues used an SLE mouse model to show that Treg cells directly regulate the severity of SLE via aberrant production of autoantibodies [65]. Another research group showed that both induced (ex vivo) Treg cells and naturally-occurring Treg cells suppress B cell activation and differentiation in vitro, as well as decreasing autoantibody secretion by B cells in a lupus mouse model [66]. Zhao et al. showed that activated Treg cells suppress B cell proliferation [67]. Interestingly, Treg cells not only suppress B cell function but also selectively kill antigen presenting B cells [67] or induce their apoptosis [65].

Recent studies suggest that MDSCs expand Treg cells [7,68,69,70,71]. Serafini et al. found that Treg cell proliferation and tumor-induced tolerance in antigen-specific T cells are abrogated when MDSC function is inhibited in vivo and in vitro respectively [68]. In addition, in vitro experiments showed that MDSCs increase the number of pre-existing Treg cells rather than converting naïve T cells into Treg cells; these findings were confirmed in an in vivo model [68]. Huang and colleagues showed that MDSCs induce development of FoxP3^+^ Treg cells in vivo [69]. In addition, another research group showed that M-MDSCs participate in Treg cell development and induce transplantation tolerance in both mice [70] and humans [71]. Taken together, these studies infer that MDSCs suppress B cell function indirectly by inducing Treg cell expansion.

## 7. MDSC Signaling Pathways Involved in B Cell Regulation

### 7.1. TNF-α Signaling

Tumor necrosis factor (TNF) is an important regulator of the tumor microenvironment [72]. There are two different types of TNF-α: transmembrane TNF-α (tmTNF-α) and secretory TNF-α (sTNF-α). The latter is a cleaved form of tmTNF-α and has distinct biological activities [73]. In addition, there are two types of TNF receptor (TNFR): TNFR1 and TNFR2. TNFR1 is expressed ubiquitously and contains a death domain; thus, signaling via TNFR1 induces apoptosis. TNFR2 is expressed only by immune cells, and signaling via TNFR2 promotes cell survival [74,75].

Xu et al. suggested that MDSCs are affected by tmTNF-α expressed on the B cell surface [76]. In addition, they found that MDSCs activated by tmTNF-α induce splenic B cell proliferation, along with differentiation of B cells into IgA-producing plasma cells [76]. This novel dialog between MDSCs and B cells takes place in the germinal center with the spleen, where MDSCs accumulate in response to the TNF signal. However, they do not describe the mechanism by which MDSCs regulate B cells; rather, they suggested the possibility of reverse signaling between TNFR2 on MDSCs and tmTNF-α on B cells, although this requires confirmation in further studies [76]. Nevertheless, many reports investigated expression of TNFR2 by MDSCs, which makes it possible to speculate about the underlying mechanism [72,75,77,78].

**Figure 3:**
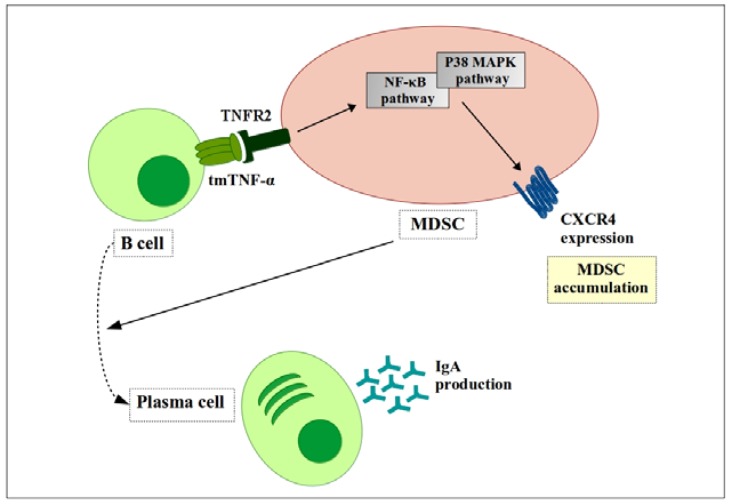
Tumor necrosis factor (TNF) signaling in MDSCs inhibits B cell maturation. Transmembrane TNF-α (TmTNF-α) expressed on the B cell surface transfers the signal to TNF receptor 2 (TNFR2) on MDSCs. That signal increases expression of CXCR4 on the MDSC surface, which drives MDSCs to accumulate at tumor sites. These accumulated MDSCs promote maturation of B cells into IgA antibody-producing plasma cells. 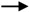, positive effect; 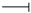, negative effect; 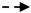, differentiation.

Zhao et al. showed that TNFR2 signaling blocks apoptosis and promotes survival and accumulation of MDSCs [72]. In addition, Polz and colleagues report the importance of TNFR2 in the suppressive activity of MDSCs [77]. Similarly, another group showed that tmTNF-α-induced MDSC activation is mediated by the nuclear factor kappa-light-chain-enhancer of activated B cells (NF-κB) or p38 pathways [78]. Later, the same group published results showing that those pathways upregulate chemotaxis of MDSCs, leading to accumulation of MDSCs in spleen or tumor tissue, and upregulate its suppressive functions [75] (Figure 3).

### 7.2. Stat3 Pathway

Stat3 plays an important role in regulating inflammation and tumor progression [9]. Among all STATs, activation of STAT3 is most effective at inducing, expanding, activating, and suppressing the function of MDSCs [79]. In response to various cytokines, phosphorylated (active) Stat3 serves as a transcription factor, promotes antitumor responses, and promotes development and recruitment of tumor-associated macrophages and MDSCs [9]. Using genetic deletion of Suppressor of cytokine signaling 3 (SOCS3), which is a negative regulator of the STAT3 pathway, Yu et al. reported that STAT3 plays an important role in tumor growth by increasing the number of MDSCs in the tumor microenvironment and by reducing CD8^+^ T cell infiltration into tumors [80].

Calcium binding pro-inflammatory protein S100A9 is a molecule that acts downstream of the STAT3 signaling pathway [9,79]. Overexpression of S100A9 promotes MDSC formation and other immunosuppressive functions [9]. In addition, the DAMP heterodimer, S100A8/A9, induces production of ROS by myeloid cells by forming a NADPH oxidase complex [9]. Furthermore, in the bone marrow, the heterodimer also contributes to development of myelodysplastic syndrome (MDS) (a B cell malignancy) by interacting with CD33 [81]. To be specific, the S100A8/A9 heterodimer is released from the bone marrow and binds to CD33 on the MDSC surface, thereby inducing secretion of suppressive cytokines IL-10 and TGF-β [81]. In addition, MDSCs contribute to development of MDS via this pathway [81].

### 7.3. TGF-β Signaling

**Figure 4:**
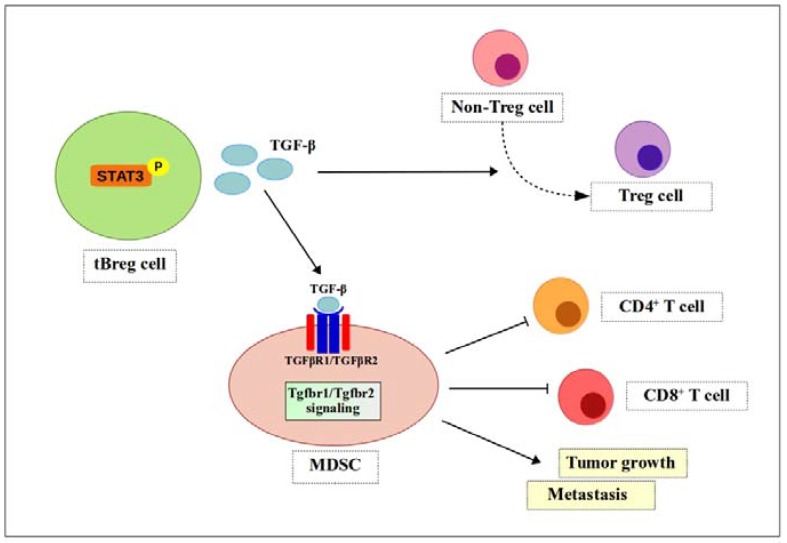
TGF-β signaling enhances MDSCs function. Phosphorylated Stat3 induces secretion of TGF-β by tBreg cells. TGF-β then binds to receptors (TGFβR1/TGFβR2) to generate fully functional MDSCs. Fully functional MDSCs suppress CD4^+^ T cells and CD8^+^ T cells and promote tumor growth and metastasis. 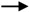, positive effect; 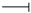, negative effect; 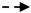, differentiation.

A report shows that CD19^+^CD25^+^ tumor-evoked Breg (tBreg) cells induce TGF-β-dependent conversion of CD4^+^ T cells to Treg cells in breast cancer [82]. To test this, the authors cultured non-regulatory CD4^+^ T cells with anti-CD3/CD28 antibodies and IL-2, together with tBregs or control B cells. They found that a significant number of non-Treg cells expressed FoxP3, a Treg cell marker [82]. TGF-β is a cytokine expressed by most immune cells; it has various functions, including regulation of inflammatory responses [9]. Bodogai and colleagues used a TGFβR1 inhibitor and tumor-bearing TgfβR2 knock-out mice to test TGF-β signaling. They found that inhibiting either one of these receptors blocked MDSC function [83]. Thus, TGF-β produced by tBreg cells not only promotes generation of FoxP3^+^ Treg cells [82] but also fully activates the regulatory function of MDSCs (i.e., increased ROS and NO production), suppresses CD4^+^ and CD8^+^ T cells, and promotes tumor growth and metastasis [83]. Moreover, TGF-β signaling also drives MDSCs to differentiate into pro-tumorigenic terminally-differentiated myeloid mononuclear cells, which contribute to angiogenesis, immune suppression, and tumor progression [84] (Figure 4).

## 8. Conclusions

Many studies have examined MDSC-mediated immune suppression. MDSC-mediated immunomodulation is directed mainly at T cells; their role in modulating B cell responses is still poorly understood. MDSCs regulate B lymphopoiesis, antibody production, proliferation, and function. Therefore, it is crucial to understand the mechanisms by which MDSCs mediate these regulatory roles if we are to develop new therapies.

Adipocyte-derived factors such as fatty acids, free cholesterol, ceramides, and lipid crystals increase production of IL-β by MDSCs, which inhibits B lymphopoiesis [29,31]. In addition, effector molecules expressed by MDSCs, including arginase-1 [33], PD-1/PD-L1, IL-10, indoleamine-pyrrole 2,3-dioxygenase [36], NO [17,37], ROS [43], TGF-β [17], PGE2 [16,48], and cysteine [17,49], all of which suppress T cell function, also inhibit B cell responses. In addition, MDSCs act on B cells directly to reduce IgM and IgG production (thereby reducing levels in serum) via VISTA [54,56], or through cell-to-cell contact [53]. By contrast, they regulate B cells indirectly by promoting expansion of Breg cells [59,61,85] or Treg cells [64,65,66,67]. Moreover, there is evidence that MDSCs modulate B cells or B cell function via various pathways, including TNFR2 [76], STAT3 [80] and TGF-β signaling [83,84,86].

Even though we have summarized the interaction between B cells and MDSCs in specific models, it is still unclear whether the identified mechanisms apply to all instances in which MDSCs regulate B cells. In addition, MDSC-mediated regulation of the function of each B cell subset has not been studied. In addition, the mechanisms underlying differences in which PMN-MDSCs and M-MDSCs regulate B cells are unclear. Future studies should address these unresolved issues.

To summarize, MDSCs are potential therapeutic targets that can be manipulated to regulate B lymphopoiesis and B cell responses and function in the context of tumors and autoimmune diseases. In addition to MDSCs, recent studies highlight the importance (beyond antibody production) of B cells during immune modulation. Considering the huge potential of B cells and MDSCs, further studies are needed to examine MDSC-mediated suppression from a therapeutic perspective.

## Abbreviations

Breg	Regulatory B cell
CIA	Collagen-induced arthritis
DC	Dendritic cell
iNOS	Inducible nitric oxide synthase
MDSC	Myeloid-derived suppressor cell
M-MDSC NF-κB	Monocytic myeloid-derived suppressor cell Nuclear factor kappa-light-chain-enhancer of activated B cells
NK	Natural killer
NO	Nitric oxide
PD-1	Programmed cell death protein 1
PD-L1	Programmed cell death ligand 1
PGE2	Prostaglandin E2
PMN-MDSC	Polymorphonuclear myeloid-derived suppressor cell
PNT	Peroxynitrite
ROS	Reactive oxygen species
SLE	Systemic lupus erythematosus
SOD	Superoxide dismutase
Stat	Signal transducer and activator of transcription
sTNF	Secretory tumor necrosis factor
tBreg	Tumor-evoked regulatory B cell
TGF-β	Transforming growth factor-beta
tmTNF	Transmembrane tumor necrosis factor
TNF	Tumor necrosis factor
TNFR	Tumor necrosis factor receptor
Treg	Regulatory T cell
VISTA	V-domain immunoglobulin suppressor of T cell activation
